# The Survival of a 580 g Infant Conceived by *In vitro*

**Published:** 2011-09-23

**Authors:** Marzieh Shiva, Mohammad Reza Shiva, Ladan Mohammadi Yeganeh

**Affiliations:** 1 Department of Endocrinology and Female Infertility, Reproductive Biomedicine Research Center, Royan Institute for Reproductive Biomedicine, ACECR, Tehran, Iran; 2 Department of Neonatology, Roointan-Arash Maternity Hospital, Tehran University of Medical Sciences,Tehran, Iran

**Keywords:** Extremely Low Birth Weight, Intra Uterine Growth Retardation, IVF, Preeclampsia, Preterm Birth

## Abstract

With recent improvements in maternal fetal medicine and neonatal intensive care, the survival rates of extremely low birth weight infants have been improved. In this report we describe the case of an extremely low birth weight infant due to preeclampsia, who was conceived by *in vitro* fertilization and is in complete physical and mental health after a one - year follow - up.

## Introduction

Preeclampsia is a multiple organ system disease that is unique to pregnancy and is often associated with significant maternal and neonatal mortality and morbidity (1). The vasoconstrictive effects of preeclampsia and perfusion reduction lead to fetal growth restriction, reduced amniotic fluid volume and an inability to tolerate the in utero environment (2). Therefore, termination of pregnancy, regardless of gestational age is often necessitated and would be the definitive treatment of preeclampsia, which results in the delivery of low birth weight babies (3).

Since women who conceive using assisted reproductive techniques may undergo personal suffering, high financial burdens and psychological stress, the preservation of the fetuses or infants in good physical and psychological health would be a vital issue for these challenged patients. On the other hand, with recent advances in maternal-fetal medicine and enhanced neonatal intensive care, the survival rates of the extremely low birth weight infants have been improved (4).

Here we report the case of a very low birth weight infant due to preeclampsia, who was conceived by *in vitro* fertilization in the Royan Infertility Centre, Tehran, Iran, and is in complete physical and mental health after a one-year follow-up.

## Case report

A 33 year-old woman with a history of primary infertility was referred to Royan Infertility centre, Tehran, Iran. Evaluation of her partner confirmed azospermia, which led to testicular sperm extraction (TESE) and diagnosis of male factor infertility. The woman then underwent an IVF trial.

The first *in vitro* fertilization (IVF) cycle was started using a GnRH-a long protocol (GnRH-a combined with Gonal F). Follicle number and growth were monitored by transvaginal ultrasound scans. Oocyte retrieval was performed transvaginally and nineteen oocytes were obtained. Nine oocytes were fertilized using intracytoplasmic sperm injection (ICSI). Ultimately three embryos at the four-cell stage, AB grade, were transferred into the uterine cavity and the remaining embryos were cryopreserved. An ultrasound scan was performed 32 days after embryo transfer and a gestational sac with a regular fetal heart rate was seen.

Two months later the next ultrasound evaluation at 15 weeks gestation revealed a fetus with two weeks’ growth retardation, severe reduction of amniotic fluid, and a 29 mm cervical canal length was recorded. Meanwhile, due to male sperm analysis results which demonstrated azospermia and a history of two mentally retarded children from the man’s previous marriage, the woman underwent amniocentesis for chromosomal evaluation and a normal 46xx fetus was recorded.

At 27 weeks of gestation, she was referred to the hospital with chief complaints of high blood pressure (180/110 mmHg), edema of the lower extremities (++), proteinuria (++) and sudden weight gain. She was diagnosed with severe preeclampsia and fetal growth retardation was noted. She received routine therapies (magnesium sulfate and antihypertensive medications including Methyldopa, Hydralazine and Labetalol) and with respect to anticipation of preterm delivery, four doses of betamethasone were given to enhance fetal lung maturity.

Finally, the patient underwent an elective cesarean delivery at 27 weeks’ gestation and a 580 g female infant with Apgar scores three and five at one and five minutes, was born. The infant was admitted to the Neonatal Intensive Care Unit and underwent intubation and mechanical ventilation for poor respiratory effort. Then she was prescribed one dose of Survanta for lung maturity improvement and after 24 hours, the respirator was separated. She was administered Ampicillin and Amikacin but due to leukocytosis and positive C-reactive protein (CRP) on day three, Vancomycin and Cefotaxime were replaced. She also received 400 mg/kg intravenous immunoglobulin (IVIG) daily for three consecutive days and the results of complete blood count (CBC) and CRP tests were maintained within the normal ranges.

The echocardiogram on day three revealed evidence of patent ductus arteriosus (PDA), which resulted in an occasional drop in heart rate. With administration of oral Ibuprofen, improvement was achieved as observed in the next echo. The cranial ultrasound scan was normal in the first week of life and no evidence of intraventricular haemorrhage (IVH) or other pathology was found. Ophthalmologic examination at the age of one month did not show evidence of retinopathy of prematurity (ROP).

Three months after birth, the infant was discharged healthy. All examinations of the neonate were recorded as normal without any complications such as ROP or IVH. The infant was in complete physical and mental health, weighing 7800 g and all developmental skills were age-appropriate after a one-year follow-up.

## Discussion

Very low birth weight (VLBW) infants are at increased risk of growth failure with uncertain prognosis after discharge from the Neonatal Intensive Care Unit (NICU) because they frequently have a protracted and complicated neonatal course (5).

According to initial reports regarding trends in mortality and morbidity for very low birth weight infants between 1991 and 1999, there have been major changes in both obstetric and neonatal care during the 1990s. These changes were associated with significant decreases in mortality and pneumothorax between 1991 and 1995. From 1995 to 1999, mortality remained relatively constant, whereas pneumothorax rates actually increased. IVH also decreased significantly over the nine year period (6). Similarly, Meadow et al. reported better survival rates in extremely low birth weight infants in the 1990s compared to the previous decade (7). Wilson-Costello et al. (4) reported improved neurodevelopment outcomes for extremely low birth weight infants between 2000 and 2002.

Meanwhile, case reports of extremely low birth weight infants were published in 1992, 1993 and 2001, which indicate that the survival of infants with birth weights below 400 g is possible without profound handicapping (8-10). A recent study in Japan also showed definite improvement in the mortality rates of extremely low birth weight infants who were born in 2005 (11).

However, the evolution of antenatal and neonatal intensive care therapy including antenatal or postnatal steroid use and new methods of respiratory support, has resulted in dramatic improvements in the survival of extremely low birth weight infants (4, 12).

Since preterm birth and delivery of low birth weight infants is mostly inevitable in preeclamptic mothers, considerable attention to these mothers and babies is essential to decrease the mortality and morbidity rates. It is noteworthy that with current improvements in neonatal care and medicine, survival of the EXLBW infants has been more achievable. However, conduction of further prospective studies, particularly regarding the longterm follow-up of extremely low birth weight infants and evaluation of subsequent cognitive abilities at school age, is recommended.
